# Knowledge engineering tools for reasoning with scientific observations and interpretations: a neural connectivity use case

**DOI:** 10.1186/1471-2105-12-351

**Published:** 2011-08-22

**Authors:** Thomas A Russ, Cartic Ramakrishnan, Eduard H Hovy, Mihail Bota, Gully APC Burns

**Affiliations:** 1 Information Sciences Institute, University of Southern California, Marina del Rey, CA, USA; 2 Brain Architecture Group, University of Southern California, Los Angeles, CA, USA

## Abstract

**Background:**

We address the goal of curating observations from published experiments in a generalizable form; reasoning over these observations to generate interpretations and then querying this interpreted knowledge to supply the supporting evidence. We present web-application software as part of the 'BioScholar' project (R01-GM083871) that fully instantiates this process for a well-defined domain: using tract-tracing experiments to study the neural connectivity of the rat brain.

**Results:**

The main contribution of this work is to provide the first instantiation of a knowledge representation for experimental observations called 'Knowledge Engineering from Experimental Design' (KEfED) based on experimental variables and their interdependencies. The software has three parts: (a) the KEfED model editor - a design editor for creating KEfED models by drawing a flow diagram of an experimental protocol; (b) the KEfED data interface - a spreadsheet-like tool that permits users to enter experimental data pertaining to a specific model; (c) a 'neural connection matrix' interface that presents neural connectivity as a table of ordinal connection strengths representing the interpretations of tract-tracing data. This tool also allows the user to view experimental evidence pertaining to a specific connection. BioScholar is built in Flex 3.5. It uses Persevere (a *noSQL *database) as a flexible data store and PowerLoom^® ^(a mature First Order Logic reasoning system) to execute queries using spatial reasoning over the BAMS neuroanatomical ontology.

**Conclusions:**

We first introduce the KEfED approach as a general approach and describe its possible role as a way of introducing structured reasoning into models of argumentation within new models of scientific publication. We then describe the design and implementation of our example application: the BioScholar software. This is presented as a possible biocuration interface and supplementary reasoning toolkit for a larger, more specialized bioinformatics system: the Brain Architecture Management System (BAMS).

## Background

**Figure 1:**
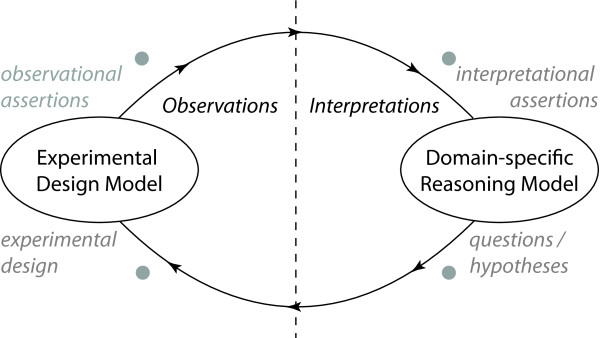
**Cycles of scientific investigation**. Scientific reasoning forms a cycle with experimental design and domain specific reasoning influencing each other. Data from experiments leads to the formation of domain theories which in turn generate hypotheses that are tested in new experiments.

The term *nanopublication *refers to a citable unit of published knowledge that refers to *a scientific assertion *with accompanying provenance metadata that permits a reader to understand where the assertion was made (author, source, format, *etc*.) [1,2]. An example of such an assertion 'Hippocampo-hypothalamic connections: origin in subicular cortex, not ammon's horn' was unusually made in a paper's title in [3], describing the localized origin of neuroanatomical projections from the hippocampal formation to the hypothalamus. If all scientific claims could be made as succinct, citable, computable elements (with appropriate justification from data suitably attached), then the thread of a scientific argument could be made by linking these claims rather than citing documents that act as their containers. This model is the goal of researchers developing representations of scientific discourse [4,5] and we present here a formulation for scientific reasoning based on experimental data within such a framework. As a central part of our formalism, we distinguish between *observational assertions *(based on specific data from carefully-planned experiments) and *interpretational assertions *(based on a higher-level understanding of the phenomena under study). This is illustrated in Figure 1 as a depiction of the reasoning process that underlies scientific research involving a direct interplay between data (observations) and theory (interpretations). We postulate knowledge constructs for each type of assertion: the 'Experimental Design Model' (describing experimental design, data and assertions) and the 'Domain-specific Reasoning Model' (describing knowledge within a subject that enables scientists *to make predictions that may be tested experimentally*). This 'Cycle of scientific Investigation' (CoSI) itself has several stages. (1) A scientist uses their knowledge within a specific domain to generate a testable hypothesis. (2) the scientist must formulate an experimental design that tests this hypothesis. (3) Having performed the experiment, the scientist may then construct *observational assertions *based on experimental data. (4) Having then interpreted (and aggregated) observations from multiple experiments, the scientist would then generate *interpretive assertions *that contextualize the data into the broader context of an underlying factual statement or claim. (5) Finally, these new revised or reaffirmed assertions may then be incorporated into the body of knowledge pertaining to the domain and may then contribute to subsequent hypotheses, *etc*. See Figure 2 from [6] for another depiction of scientific investigation as a cyclic process. Within this paper, we describe a formulation called '**K**nowledge-**E**ngineering **f**rom **E**xperimental **D**esign' (KEfED) and then demonstrate the ability to generate and reason over interpretive assertions within a well-defined scientific domain. Neural Connectivity (the study of connections in the brain) has been popular within the field of neuroinformatics for roughly two decades. See [7] for an seminal paper deriving a hierarchical processing scheme for cortical areas in the Macaque based on the laminar patterns of origin and termination of cortico-cortical connections. Work has involved the development of connectivity repositories [8-11], mathematical analyses [12-15] and high-level theories of brain organization [16] based mostly based on neuroanatomical tract-tracing studies in animal subjects. These studies involve injecting a minute quantity of tracer chemical into a structure in the brain. This tracer is taken up by neurons that impinge upon the injection site and then transported along the neurons' axonal fibers (either from a neuronal population's cell bodies to their axonal terminals for *anterograde *tracers or from axonal fibers to the cell bodies for *retrograde *tracers). By processing and examining the tissue histologically, it is then possible to infer the existence of neural projections between the location of the injection site and the location of transported label [17].

**Figure 2:**
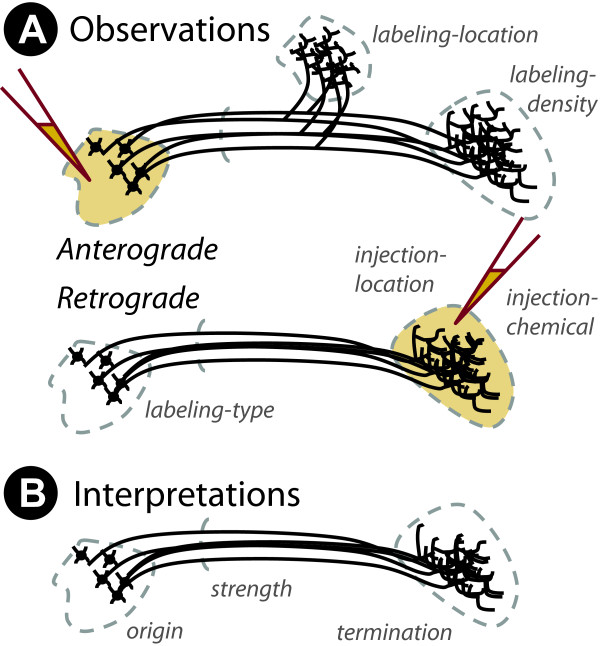
**Basic reasoning model**. The basic formulation of our tract-tracing reasoning model: (A) structured observations within tract-tracing experiments based on the experimental parameters and measurements that describe anterograde and retrograde experiments and (B) the interpretation of this data to describe connections between brain structures.

This relatively simple experimental design provides a concise demonstration of the CoSI model. Tract-tracing experiments simply consist of a surgical injection of a chemical to a targeted location in the brain, followed by histological processing and neuroanatomical analysis. For this information to become a description of neural projections between brain structures, knowledge of the uptake and transport properties of the tracer chemicals must be invoked (see Figure 2). Thus, *observational assertions *should be formulated without background knowledge (save that required to define terminology unambiguously) and *interpretational assertions *invoke background knowledge to generate a knowledge base. It is important to note, that if the background knowledge changes then so too do the interpretations.

The formulation of the KEfED model for tract-tracing experiments focuses on measurements of the ordinal labeling density ('none', 'sparse', 'moderate', 'dense') Although in some rare cases, retrograde studies may be quantified through the use of careful cell counts, this is rarely reported. We only use ordinal scales in order to maintain a tractable, uniform approach. and labeling type ('cells', 'fibers') of the transported tracer indexed by parameters pertaining to (a) the location of the injection site defined by reference to a well-defined neuroanatomical nomenclature, (b) the tracer chemical used, (c) the locations surveyed for transported label (Figure 2A). These five quantities are sufficient to generate an interpretation asserting that there exist neurons in a region of origin that project to a region of termination with a specified connection strength (Figure 2B) [18].

At a high level, we capture the primary experimental observations of these experiments as parameters, constants and measurements (the location of the injection site, the tracer chemical, the location, type and density of transported labeling). The interpretations that contribute to a model for reasoning about neural connectivity would be simply the locations of both a given projection's origin and termination and perhaps its *strength *(which would take the ordinal values: 'none', 'weak', 'moderate', and 'strong').

This is the coarsest possible reasoning model of neural connectivity ('macroconnections' or gross-level projections between named grey matter volumes in the brain) and it is a prominent goal of the community to develop finer-grained representations (either 'mesoconnections' at the level of cell populations or 'microconnections' at the level of individual neurons) [19]. Other new methods of data acquisition are responsible for generating a great deal of new interest in studying 'connectomics' [20]. These methods include Functional Magnetic Resonance Imaging and Diffusion Weighted Imaging for gathering neural connectivity data in humans [21]. There are also data-intensive methods to examine *all *synaptic connections between a small number of neurons within a very small volume of neural tissue through serial reconstruction of electron micrographs [22,23].

Despite these methodological developments in the field, our focus in this paper is concerned with using an example data set that demonstrates the *interplay between a specific experimental design model and its derived interpretation*. We assert that tract-tracing experiments provide the best-quality data for neural connectivity in non-human species and so are the best candidates for developing this model. As a software-based study, we present a working implementation of this software, instantiated as a read-only demonstration for neural connectivity (accessible via our project website: http://www.bioscholar.org/) and as a fully-functional editable system, open for use in other domains (accessible via our development website: http://code.google.com/p/bioscholar/).

## Implementation

BioScholar has both a general, domain-independent component and a customized domain-specific reasoning component. The KEfED editor with its associated experimental designs do not depend on a particular scientific domain. They can be used to represent and store scientific experiments in any domain, and are not limited to tract-tracing or neurobiology. KEfED models and the data from associated experiments can be stored and manipulated using the BioScholar program without any customization. Reasoning models and queries for interpreting the data from an experiment are domain-dependent, almost by definition. As a case study, we present tract-tracing experiments and the derivation of a matrix showing brain region connections. The computation of the *connection matrix*, along with the geometric reasoning that form the neuroanatomical parts of BioScholar use additional resources such as brain atlases and background knowledge about the tract-tracing methodology. These domain-specific reasoning models are specifically designed to use data from a specific experimental model. Such reasoning models operate on the measurement variable values and their associated context to generate suggestions of evidence and tentative conclusions based on the underlying scientific theories that inform the creator of the interpretation. This part of the BioScholar is, therefore highly customized for a particular application.

The downloadable software includes the generic BioScholar application and a specific neural connectivity demonstration. The generic BioScholar application can be applied to any domain and provides a graphical editor for experimental designs and a storage system for experimental data. The neural connectivity demo adds a domain-specific panel to the BioScholar application that displays the connetion matrix for the hippocampal region of the brain and can show the underlying studies for each matrix entry.

### KEfED Models of Tract-Tracing Experiments

KEfED models are composed of experimental variables: either *parameters or constants *that are predefined as part of the experimental design (and either vary within the experiment or are held constant); or *measurements *that form the primary data from the experiment. Our central premise is that observational assertions are typically based on the statistics of the measurements made within an experiment. Each measurement has a *context *provided by the set of parameters that describe the conditions under which the measurement was made.

**Figure 3:**
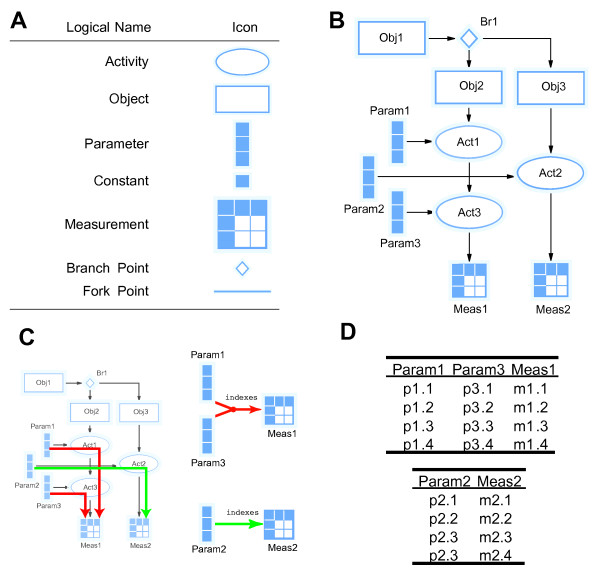
**Components and mechanisms within the KEfED model**. (A) Each model is made up of these elements (Activities, Objects, Variables and control flow elements). (B) A hypothetical example: showing multiple Objects, Activities and Variables. (C) The dependencies of variables in [B] based on pathways through the protocol. (D) Tabulated hypothetical data for the two measurement variables in this example.

The indexing mechanism used to generate the context that links parameters to measurements is based on a workflow representation of the experimental protocol. We construct a graph representation of *experimental objects, activities *(that act on the objects, possibly transforming them into other objects), *branches *and *forks *(that allow the workflow to divide), *parameters, constants *and *measurements*. This overall methodology is illustrated in Figure 3. The indexing of a measurement is based on a path through the workflow back to the starting point of the protocol's workflow so that any parameter or constant falling on this path is used as an index (see Figure 3B,C &3D). This intuitive methodology provides a powerful basis for practical knowledge engineering technology.

**Figure 4:**
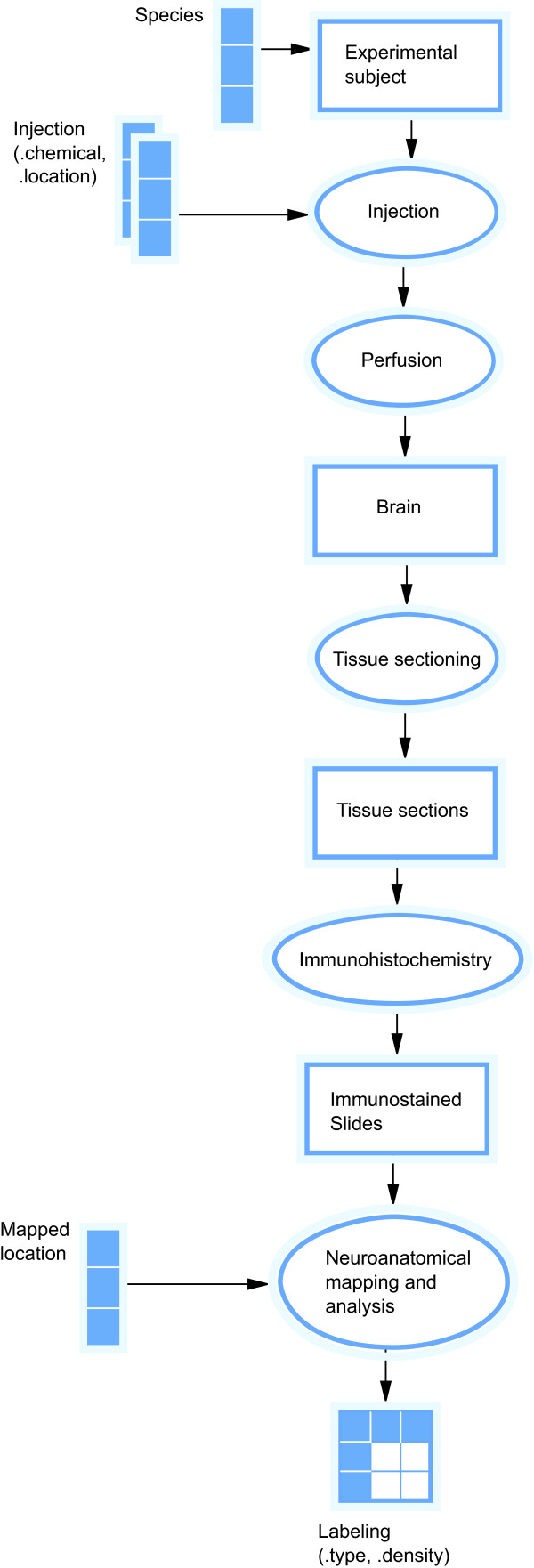
**KEfED model for a tract-tracing experiment**. A KEfED model for a generic tract-tracing experiment as shown in the KEfED editor. The workflow provides a high-level view of the provenance over the course of the complete experimental protocol starting with experimental subject (in this case, a rat) through the material processing that generates experimental material that may be analyzed to generate data (in the form of an account of the presence of histological labeling of varying density and type at different locations in the brain).

We have constructed a KEfED model for tract-tracing experiments (see Figure 4) which forms the basis of our demonstration application. We offer preliminary definitions for both the variables and other elements of the model (see Tables 1 and 2). The KEfED editor can currently annotate model elements (experimental objects, activities and variables) with terms from external ontologies. We invoke an intermediate-level representation of the experimental protocol where each step of the process is represented coarsely. For example, the procedure of performing a precise stereotactic microinjection of tract-tracer chemical is represented with a single model element (an 'Injection' activity) with two attached parameters (the location of the injection site and the type of tracer chemical injected).

**Table 1:** Definitions of KEfED variables for tract-tracing experiments

Variable	Definition
Taxonomic class	Within this example we adopt the designation of taxonomic Specification from the NCBI's taxonomy database [63].

Injection.chemical	The chemical molecule injected into the brain of an animal as part of a tract-tracing experiment. These reagents are not confined to a specific type of chemical simply have the property of when injected into brain tissue, they are taken up and transported along axons by active processes in neurons. Early examples of these include tritiated amino acids [64], Horseradish peroxidase (HRP) [65] and notably Phaseolus *Leuco-Agglutinin *[66]. Tracer chemicals include fluorescent dyes and beads and even include more exotic materials such as viruses and Cholera Toxin. See [17,67-71] for reviews of the general and specialized methods used.

Injection.location	This refers to the extent of the complete injection site expressed in terms of its spatial relationships to identified brain structures from the Swanson atlas [32] and encoded into the neuroanatomical nomenclature specified from the BAMS system [72]

Mapped location	Similarly to Injection.location, this refers to the spatial extent of an individual region of labeling (judged by the scientist reporting the study to be relatively homogeneous) expressed in terms of its spatial relationships to named neuroanatomical terms from the BAMS ontology [72]

Labeling.type	A simple categorization of the part of the neuron that is labeled. This is a nominal data element with possible values 'cellular' (denoting that the neuronal cell bodies were labeled), ' fibers' (denoting that axonal fibers were labeled), or 'terminals' (denoting that axonal fibers with characteristics of a terminal region were labeled, including heavy branching and the presence of boutons).

Labeling.density	A simple, seven point ordinal scale with the following categories (in order): 'no label', 'very sparse label, 'sparse label', 'sparse/moderate label', 'moderate label', 'moderate/dense label' and 'dense label'. We also include an additional category to denote that labeling is present but with an unknown density.

Definitions for the variables used in the tract-tracing study. The KEfED system allows for complex multi-attribute variables (such as Injection.chemical and Injection.location).

**Table 2:** Definitions of KEfED processes and entities for the tract-tracing experiment workflow

Element	Definition	Ontology term
Experimental Subject	The living organism that is the subject of the experiment.	obi:OBI_0100026 ('organism')

Injection	A microinjection of tracer into the brain of the experimental subject.	obi:OBI_0000426 ('injection')

Perfusion	Euthanizing, exsanguinating, and then perfusion-fixing the tissue of an experimental animal. In our case, we also include in this step the process of tissue dissection that extracts the brain from the body and preserves it (by freezing) for subsequent histological processing.	obi:OBI_0000919 ('animal euthanization')

Brain	The post-mortem dissected brain of an experimental subject	fma:FMA_50801 ('Brain')

Tissue Sectioning	The process of cutting a biological sample (in our case, a dissected brain) into thin tissue sections for histological staining and processing.	nif:birnlex_2156 ('Tissue sectioning')

Tissue Sections	Unmounted thin sections of tissue (thickness typically less than 100 microns) for subsequent histochemistry, staining and mounting	nif:birnlex_2169 ('Tissue section')

Immunohisto-chemistry	The process of histochemistry, staining and mounting of tissue sections onto microsope slides.	nif:nlx_inv_20090609 ('Immunohistochemistry')

Immunostained Slides	Thin sections of tissue that have been mounted on glass slides for subsequent microscopic examination and analysis	-

Neuroanatomical mapping and analysis	The process of microscopically examining neuroanatomical sections in order to place accurately the location of histological staining into the context of a standard brain atlas/parcellation scheme.	obi:OBI_0600020 ('histology')

KEfED model elements for the processes and entities in the tract-tracing experiment, showing the closely matched terms from community-driven ontologies. Those terms were added to the KEfED model elements using the editor's ontology search interface. Sources of the terms are the Ontology of Biomedical Investigation ('obi: http://purl.obolibrary.org/obo/', [44]), the Foundational Model of Anatomy ('fma: http://purl.org/obo/owl/FMA#', [73]), the Neuroscience Information Framework ('nif: http://ontology.neuinfo.org/NIF/DigitalEntities/NIF-Investigation.owl#', [74]). The matched term is intended to be as close a semantic match as possible. If a specialized term is not available, a more general encompassing term is used.

### KEfED and Geometric Reasoning

We perform our reasoning using the PowerLoom^® ^first-order logic knowledge representation and reasoning system [24]. PowerLoom provides us with a deductive reasoning engine that supports numerical calculations, *n-ary *relations and closed-world reasoning. PowerLoom has been developed over the course of ten years and applied in numerous domains including hybrid reasoning systems [25,26], natural language understanding [27], metadata search [28] and interest matching [29]. It has a query language that allows us to access the information from our encoding of the experimental structures. We use the Java implementation of PowerLoom, which also has support for a web services interface that we use to integrate our KEfED reasoning system. We use queries and inference rules to construct interpretable statements concerning the existence and strength of connections between brain structures based on KEfED-based assertions. Not all of the additional expressive power of PowerLoom is used in the neural connectivity example. However, we do take advantage of the ability to create defined properties and define n-ary properties that can be used in constructing complex queries over the data. So that, for example, if we wanted to understand projections from the Postsubiculum (POST) to the Retrosplenial (RSP) area, the system would construct queries for experiments where injections of anterograde tracer were made into POST and terminal labeling was found in RSP *or *injections of retrograde tracer were made into RSP and cellular labeling was found in POST (see [30] for an example of this experiment).

This reasoning system also provides support for reasoning about *geometric relationships *between different brain regions. In tract-tracing experiments, tracer injection sites may be reported to be within particular regions, their subregions or to overlap two or more named structures. Differences in nomenclatures across studies also may cause variation in the degree of detail use to describe which brain regions are implicated in a given experiment. Our reasoning system must therefore be able to understand the geometric relationships of these regions.

The primary relationship of interest is regional containment, *i.e*., how regions are enclosed by each other. This also allows us to aggregate information from studies that studied different subregions. We support the reasoning over a containment hierarchy through the definition of a transitive containment relationship 'PROPER-PART-OF' for denoting a spatial region which is a proper part of another region. We also use an 'OVERLAPS' relation to describe a region that covers a part of one region along with at least a part of another disjoint region. Since injected tract-tracing can often spread to adjacent brain regions, this is necessary for a proper description of the actual experimental results. When looking for injections of interest, we want to find injections into subregions of our region of interest. This is computed using 'PROPER-PART-OF' and its transitive closure. But in addition we are also interested in finding injections that overlap a subregion of our region of interest. We make use of PowerLoom's ability to define relations to craft a specialized relation that represents regions that are part of the region of interest or that overlap a region that is part of a region of interest. By creating this named relation, we are able to build a series of other relations that describe the results of anterograde and retrograde experiments in a modular manner. We have tools that import the basic geometric relationships from the brain atlases. We translate the neuroanatomical ontology for the rat provided by provided by BAMS [31] into PowerLoom where we use a transitive containment relationship to provide a hierarchy of brain regions. Details of this mapping are described in additional files linked at the end of this article, including (a) a description of the process used to import brain region containment data (Additional file 1), (b) a copy of the containment data obtained from the BAMS database (Additional file 2); (c) A set of three PowerLoom files that describe qualitative geometric relations, their use within an atlas and an instantiation of these relations for a specific neuroanatomical atlas (Additional files 3, 4 and 5) [32]. This allows us to use the reasoning system to manage the containment hierarchy and perform simple inferences on demand, in response to system queries. For our example above, we would also need to be able to retrieve KEfED assertions that involve subregions of POST or RSP. RSP contains dorsal (RSPd) and ventral (RSPv) subregions, the latter of which has additional subdivisions RSPv-a, RSPv-b/c in the BAMS neuroanatomical nomenclature [31].

### A Web-Based KEfED Curation System

We have built a prototype user interface for editing KEfED models as a Flex-based rich internet application. We used Kap-Lab's freeware (but closed-source) Diagrammer program as the basis for this tool [33]. This is a Flex component that permits users to construct graphs from elements that defined as SVG-based primitives (Figure 3A). It links these graphical elements to underlying ActionScript classes defined by external developers. As the basis for these internal data-structures, we adopted the graph-based representations from the Flare Prefuse ActionScript library, in order to use their graph-traversal and shortest-path algorithms [34]. This permitted us to implement the KEfED model entirely within the Flex interface as a web-application within an environment supplied by the Tomcat Web Server. We used the Persevere JSON-based web-accessible database to provide a generic, flexible storage for the KEfED models generated within our application [35]. Since Persevere's HTTP-based services for editing and deleting models required the use of PUT and DELETE HTTP calls, we deployed the KEfED editor web application with a proxy server based on the Adobe Blazeds messaging library. Using this application, an experimental protocol can be built up (Figure 4).

**Figure 5:**
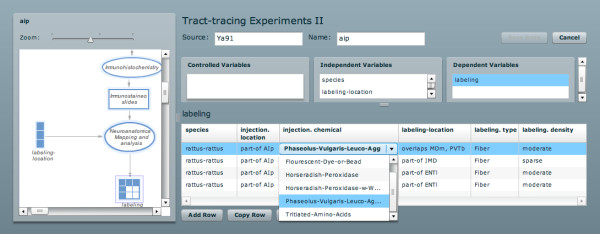
**KEfED data form for a tract-tracing experiment**. The KEfED editor showing the data form derived from the tract-tracing model. The data entry table (spreadsheet) view is generated automatically by tracing the data dependencies in the model design. This insures that the entire relevant context for measurements is captured. This form shows data entered from a curated experiment [62].

The KEfED editor uses the experimental protocol to trace data dependencies and automatically generate data input forms following the process in Figure 3. From the tract-tracing model (Figure 4) we generate an input form for recording the necessary data (Figure 5). The columns are derived by tracing the data dependencies for the measured values (labeling type and density) along the protocol to the parameters for the experiment (species, injection location and chemical and labeling location). Tracing along the dependency links assures us that the relevant context for proper interpretation of the data is preserved. Some of the parameter values are may be considered constant, either across all instances of the class of experiments (since we are only considering studies in rats, the species variable is constant) or sometimes for a particular experiment (once selected in a specific experiment, the injection chemical does not usually vary).

The spreadsheet interface uses information from the experimental design to present an appropriate interface to support data entry. Any variables with a fixed set of values result in a pop-up menu of choices for the input. Anatomical regions have a special widget that allows us to capture not only the region, but also the relationships between an arbitrarily-defined region of an injection-site or labeling-location and the named structures in the brain atlas.

### System architecture

**Figure 6:**
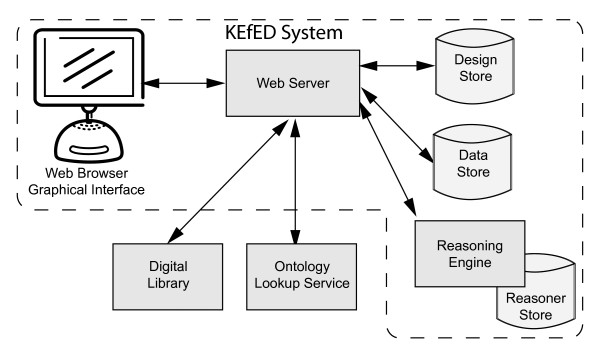
**Components of the KEfED editor system**. The user employs a web browser to interact with a Flash-based graphical interface. The interface uses a storage system, currently the Persevere noSQL database, for experimental design and experimental data storage. PowerLoom is used to provide inference services in support of interpreting neural connectivity observations. An external web service such as the NCBO BioPortal [36] provides access to ontological terms for semantic annotation of KEfED models.

A component diagram illustrates the overall system architecture with our current implementation of the KEfED editor system (Figure 6). The central hub of the system is a web-application running on an Apache Tomcat web-server. The client application is a Flex 3.5 application running through a BlazeDS remoting/messaging service on the server. This permits the client to communicate via HTTP, SOAP and REST services with external resources (such as the NCBO's BioPortal ontology repository [36], our locally-hosted digital library system and our web-service interface to the PowerLoom reasoner). We uses two server-side Persevere repositories (one for the experimental designs, one for experimental data) and a PowerLoom knowledge base.

**Figure 7:**
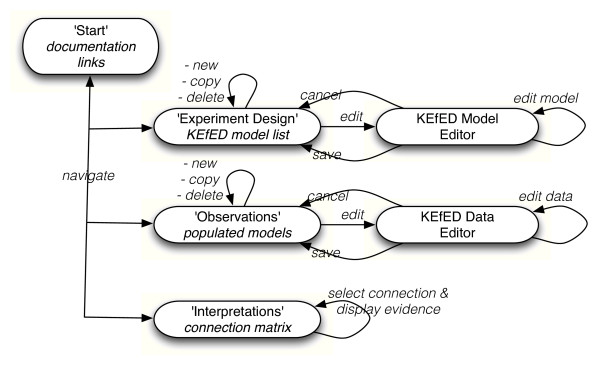
**State diagram of the BioScholar application**. These are the states and high-level activities available to users. Users may navigate between the 'Start', 'Experimental Design', 'Observations' and 'Interpretations' application views. They can create, copy and delete designs from the list of KEfED models and then edit them from within the KEfED Model Editor. Similarly, they can enter, copy or delete experimental data sets from the list of experiments and then edit data for an individual experiment using the KEfED Data Editor. Finally, they can examine a domain-specific interpretation of experimental data (for neural connectivity based on observations from tract-tracing experiments). These interpretations are based on querying the knowledge base for supporting data elements with links to the publications from which the data is drawn.

Figure 7 shows a state diagram for the current release of the system (each rounded rectangle represents a state of the system and the arrows represent system activities that may involve transitions between states [37]). The entry point is the 'Start' page that only contains hyperlinks to external pages for documentation and user feedback (the BioScholar web site, a Google code project page and an in-house wiki). At this point, the top-level controls of BioScholar are arranged in an accordion control that allows easy navigation between the 'Start', 'Experimental Design', 'Observations' and 'Interpretations' states. Each state provides specific functionality.

Within the 'Experiment Design' tab, the user is presented with a list of KEfED models. At this point they may add a new blank model, copy or delete an existing model or edit one of the models in the list. If the user chooses to edit a model, they are taken to the main KEfED model editor panel, where they may draw a model on a graphical palette. Selecting each element in the diagram, changes the available controls to edit the semantic details of that element (changing the name, editing the associated values available for a specific variable, attaching a specific ontological term to the element, *etc*.). At the global level, the user may then save or cancel their edits to taking them back to the list of all available models in the system. The 'Observations' tab allows the users to add data to a KEfED model corresponding to the execution of an individual experiment. As is the case with our representation of tract-tracing experiments, one KEfED model can provide a template description for many experiments. This component shows a zoomable navigator control that that allows to the user may use to select variables within the experimental design and edit data their data values. This allows a scientist enter both the values of measurements and their parameter-based context (see Figure 5).

**Figure 8:**
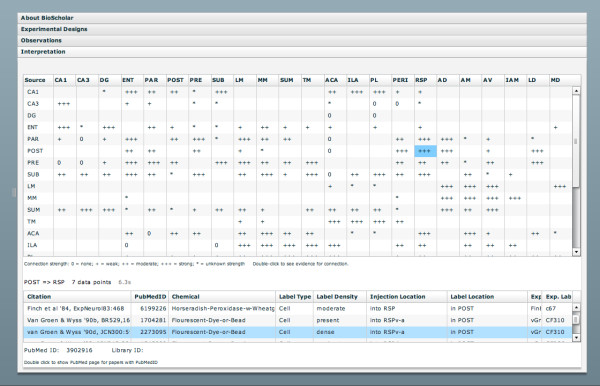
**Connection matrix**. The connection matrix shows an interpretation of tract-tracing experimental data. The upper table shows a connection matrix for brain regions in the hypothalamus. The regions use abbreviations from the BAMS nomenclature [31] and show the strongest connection for which there is some experimental evidence. Double-clicking a cell will issue a query to show the supporting evidence. This evidence is summarized in a table below the matrix. In this view, we show evidence for a connection between the postsubiculm (POST) and the retrospenial (RSP) area. The highlighted experiment involved the injection of a flourescent dye into subregion 'a' of the ventral RSP (RSPv-a). The dye moved in a retrograde manner to label cell bodies in the POST. Note that this involves containment reasoning about brain regions.

The 'Interpretations' tab will only ever be present when the system has been tailored for a specific reasoning model (since interpretations are domain-specific). In this case, the component contains a 'connection matrix' that tabulates hard-coded macroconnections that are reported in the knowledge base (Figure 8). This matrix should be considered a rudimentary reasoning model for neural connectivity. By double-clicking on a cell in the matrix, the system will issue a query to the PowerLoom knowledge base and retrieve all known observations that are relevant to the interpretation of interest. In this way, the system may directly link observational and interpretational assertions as shown in Figure 1. These observations are further linked to the underlying literature. Those that are indexed by PubMed can also have their PubMed page displayed in a separate browser window.

## Results

As the main demonstration of the feasibility of this approach, we populated the knowledge base with connectivity information from [14]. This study describes a detailed analysis of the connectional topology of neural systems associated with spatial navigation in the rat (involving the hippocampus proper, the retrohippocampal formation, the mammillary nuclei of the hypothalamus, and parts of the so-called 'limbic' cortex and thalamus) based on manually-curated connectivity data from the primary neuroanatomical research literature as part of [8]. This information was contributed to the BAMS repository and subsequently augmented with a small number of subsequently published studies. The focus of this paper is not concerned directly with making this particular knowledge base complete or up-to-date (it was originally curated in 1997), but we use it as a well-defined starting point for this current implementation. The neural connectivity results from the tract-tracing experiments includes data from 188 publications. Manual curation [10,14] divided the information into 850 experiments comprising 3,210 data points. Each data point corresponds to a relation linking an injection report to a single labeling report. An experiment corresponds to multiple data points relating to a single injection. The connection matrix is a 23 by 23 matrix of brain regions in the Hippocampus. Querying for the data elements supporting an individual connection (which involves reasoning over the PowerLoom Knowledge Base) generally executes within 10 seconds (typically from 2-5s per query). Generating the entire connection matrix takes roughly 90 minutes, and is performed off-line so that the results may be cached for display in the program. The retrieval of supporting items is performed as a live PowerLoom query using a web-service interface. Of the 3,210 data points, 1,099 reported on connections with both endpoints in the hippocampus.

Timing results are based on a Macintosh PowerBook with a dual core 2.4GHz Intel Core2duo processor with 4GiB RAM, Mac OS × 10.5.8 using the Java version of PowerLoom 3.2.52 and 64-bit Java 1.6.0_20 running with 1GiB of heap space allocated. Query results use software timing, which is reported in the interface for individual connection queries. The web browser and servers were running on the same host, which minimizes network delays.

The connection matrix is an interpretation and summarization of the underlying experimental results. The experiments report the transport of marker chemicals and dyes between brain regions. The interpretation of that transport is the fairly simple inference that the marker transport indicates a connection between neurons in the source and destination brain regions. This is made only slightly more complicated by the need to differentiate between anterograde and retrograde transport. The interpretation is computed by examining the data as represented by the model parameters and measurements. This structure is derived from the KEfED model design and insures that the appropriate context is available for interpreting the data.

In addition to making this inference, the connection matrix also provides a summary by defining the structures that frame the results. Some geometric reasoning may be needed to map from the histological observations to the connection reports. In addition, there is also the need to account for injections that spread and cover multiple brain regions, since such data provides weaker evidence for a connection because the marker chemical could have come from one of the other portions of the overlap. These elements should be included, so that an analyst can factor that into the considerations when reviewing the evidence for a particular connection.

The use of geometric reasoning is a significant contributor to the generation of the connection matrix. Out of the 1,099 individual connection reports, 290 involved the use of part-of reasoning and 440 used overlap relations. 101 our of 246 connections did not have any direct evidence and could be found only by considering the effects of geometric containment or overlap. An example of this is the connection between field 'CA1' and the entorhinal ('ENT') areas of the hypothalamus. We curated five papers reporting connections between those regions. Beckstead's paper [38] reported a reterograde study with three separate injections, one generally in ENT and the other two in specific sub regions ('ENTl' and 'ENTm'). All three showed tracer in CA1. Deacon [39] reported a retrograde study with separate injections into three regions, each of which overlapped ENT but also included other areas, with labeling in CA1. Swanson [40,41] published the results of two experiments with retrograde tracer injected into ENT and found in CA1. Finally, van Groen [42] reported an anterograde experiment with two injections into CA1 and labeling found in ENT. The geometric reasoning as well as consideration of the direction of marker transport had to be combined in order to assemble the full set of evidence for a connection between these regions.

The inferential reasoning makes use of PowerLoom's ability to define n-ary relations and provide rules for determining the values. (see the PowerLoom manual [43] for details on the language) These relations are then used to build up the queries. In effect, they can act like pre-defined queries which allow sharing of the inferences and simplify the engineering of the domain model and the resulting creation of queries. An example we use to illustrate this mechanism (shown below) is based on a relation for computing the admissible geometric relationship between injection sites and the regions of interest in the connection matrix. This involves a combination of reasoning about part-whole relationships as well as extending that reasoning to include the effects of overlapping regions. This relationship is defined in PowerLoom by

(DEFRELATION part-of-or-overlaps (?sub ?super)

   :DOCUMENTATION "Checks whether ?sub is contained in super, or whether ?sub overlaps

         with ?super, including overlapping a part of ?super"

   :<= (OR (= ?sub ?super)

      (/PART/PROPER-PART-OF ?sub ?super)

      (/PART/OVERLAPS ?sub ?super)

      (EXISTS ?overlap

         (AND (/PART/PROPER-PART-OF ?overlap ?super)

            (/PART/OVERLAPS ?sub ?overlap)))))

This definition states that the relation 'part-of-or-overlaps' is satisfied if

1. The two regions are the same or

2. The sub-region is part of the super region or

3. The sub-region overlaps the super region or

4. There is some other region that is part of the super region and the sub-region overlaps that other region.

This illustrates the expressive power of the PowerLoom language. By defining this relationship once, it can be easily re-used in various queries. Other relations are also defined with more complicated structure that are used to extract the data and properly interpret the direction of connection depending on whether an anterograde or retrograde experiment is being considered.

## Discussion

The task of curating data from literature resources is a serious challenge for developers of bioinformatics resources and, although the community lacks globally-applicable, production-level, open-source tools, there is a continuing effort to generate ontological standards, practical conventions and software to provide support. Several other efforts utilize similar constructs to KEfED in their efforts. OBI's protocol-based view of experimental design as a general ontology capturing experimental methods [44] motivates the development of several notable systems. The VIOLIN project is a web-based vaccine database and analysis system that both provides a repository for vaccine-based information and a suite of bioinformatics tools for literature mining and even the prediction of potential vaccine targets [45]. The ADAM system uses an ontological representation within a detailed conceptual model that effectively cycles through the cycle shown in Figure 1 for a well-defined domain-specific model pertaining to yeast molecular biology [46,47]. The 'ISA' family of tools [48], derived from the phrase: '**I**nvestigation, **S**tudy, **A**ssay', are based on a spreadsheet model that is similar to the KEfED representation of data.

Computational systems of scientific discourse such as SWAN (Semantic Web Applications in Neuromedicine [4,5]) and the development of the concept of *nanopublications *are particularly relevant to this effort [1,2]. As a formalism for scientific knowledge engineering, our KEfED-based toolset is significant for four reasons: (A) it is conceptually simple; (B) it is generally applicable; (C) it is comprehensible to biologists and (D) it supports a model of *scientific inference*. By developing a concrete implementation for this formalism, we not only hope to make it more accessible to end users, but strengthen our ability to study and improve the approach in collaboration with our colleagues cited above. Although we have focused primarily on the use of this methodology for literature-based curation, it may also be used to curate primary data [49].

In addition to data-driven tool development, we are also engaged in developing machine-reading tools that specifically target the definition of variables and their values to be extracted from natural language text in the published literature. The Utopia documents system uses published PDF files as a live interface over underlying semantics that could be defined in a variety of frameworks such as KEfED [50]. The goal of developing these new approaches and tools is to re-engineer the process of scientific publication, communication and discovery to leverage computable models directly into the process so that it becomes automatable and therefore scalable.

Other work on scientific workflows [51,52] uses a very similar formulation for scientific protocols. The Taverna [53,54] and myExperiment [55] systems, in particular, have been used to create and share executable workflows for biomedical applications. Development work in this field has concentrated on describing *machine-executable *workflows for data analysis. The emphasis in KEfED is on a different and more general part of the process. KEfED activities are more general in the sense that they do not require an executable computational step to be associated with them. In a typical KEfED model (as currently implemented), we do not expect to execute the protocol as data processing (especially since many of the elements represent material entities rather than information artifacts and therefore cannot be processed computationally). It is, however, an interesting future design goal to link our KEfED-based representation of the pre-computational part of a scientific workflow to executable tools that may process the data represented in the KEfED format. KEfED models could be extended to include Taverna modules as elements. and KEfED-enabled webservices could be made available as components to be used in Taverna workflows.

Part of the value of the KEfED approach is it's intuitive appeal and simplicity for biomedical experts (hence its capability of being embedded into the editor software described in this paper). KEfED models are currently composed of a relatively small number of semantic elements: (i) entities and (ii) processes involved in the experimental protocol, (iii) experimental variables that contribute to the interpretation of observations and (iv) the values of those variables. The BioScholar system currently allows entities, processes and variables to be annotated with ontology terminology via a lookup tool that uses the BioPortal web-service from the National Center for Biomedical Ontology. We provide a very small vocabulary of terms for our Neural Connectivity use-case in Table 2. The Ontology for Biomedical Investigation (OBI) is a community-driven effort to construct a well-defined formal ontology for 'the description of biological and clinical investigations' [44] based on a top level formulation provided by the 'Basic Formal Ontology' (BFO) [56]. Future work is planned to exploit the correspondence between KEfED elements and high-level classes within OBI and even to use the KEfED editor tool as a possible curation interface for ontology development within the OBI community.

Some of the reasoning processes used in the neural connectivity example could have been described using the OWL 2 [57-59] Web Ontology Language, since it provides the ability to define and reason with transitive relations. However, we found the ability to define n-ary relations and rules for inferring the values of such relations provides a software engineering advantage. We may define complicated relationships and use them as named queries to facilitate the construction of evidence for neural connectivity. We also found the existence of a built-in query language to be convienient for development. In the future, moreover, we expect to make more use of the greater expressive power of a first order language and also to make use of PowerLoom's ability to perform arithmetic computations and support extensions for the addition of statistical reasoning.

In principle, however, one could apply a number of different reasoning systems that work over data curated with the domain independent part of BioScholar. All that would be needed would be the development of appropriate export functions for saving the KEfED-curated data in an appropriate format for the reasoning engine. We plan to make export of the data in an OWL compatible format part of a future version of BioScholar.

### Future Directions

The system as it appears here is a prototype built with some non-standard elements (such as Persevere, PowerLoom, *etc*.) that will be modified going forward. We anticipate developing the KEfED methodology to be maximally compatible within the field of 'Semantic-Web' approaches to biomedical informatics, by expressing KEfED models in OWL/RDF and by improving ontology harmonization with the OBI project. In particular we will extend the ability to annotate particular variable values with ontology terms and eventually also use ontologies as the sources of variable values.

We expect to develop KEfED-driven nanopublications in the near term. We anticipate developing KEfED-based technology relatively small plugin components for other sites and systems. Given also that the main source of information currently for our work derives from the scientific literature, we are actively developing text mining tools to assist with the curation of data into KEfED models themselves [60]. As an exercise in knowledge modeling, the formulation of an individual KEfED model may be expected to evolve (for example, should the location of histological labeling be considered a parameter or a measurement? Is the location of the injection site a parameter or a measurement?) and thus, additional functionality built into the modeling software could promote and support this through a versioning function. Finally, we intend to evaluate the system from the point of view of its performance for well-defined knowledge management tasks (including a comprehensive view of evaluating the validity of the model and its usability) [61].

Future work for the KEfED formulation itself will be to (a) represent relations of statistical significance between measurements with a parameter-based measurement context and to (b) represent correlations between variables. The way that we construct the measurement context becomes more complex than our current formulation can accommodate when data are processed in a such a way as to combine or distort the role of individual parameters. For example, a parameter we might track in an experiment is the identifier of a particular experimental subject. If we calculate the statistical mean value if a measurement, then the calculation involves aggregating measurement values across all experimental subjects, thus removing the id values of each individual subject from the mean value's measurement context. In order to expand and generalize our approach, we need to capture explicitly this mechanism into the underlying design of the KEfED formalism. Other, more complex elements to be modeled and included are 'loops' within the experimental design (where an assay or processing step is repeated many times based on an indexing variable, such as time).

## Conclusions

We here present the KEfED formalism as a model for reasoning over scientific observations that support a given interpretation. We have instantiated this formalism within a general-purpose, open-source, fully-functional web-application that may be freely downloaded and used. We have provided a worked example from the domain of studying rat brain neural connectivity. The system is an early prototype but is designed to provide basic functionality to end-users and to provide a framework for future development within the field of biomedical knowledge engineering.

The functionality of the KEfED editor provides benefits at three levels of the curation process.

1. Provides a means to specify an experimental design that is intuitive for biologists to use. This design is then use to create data capture forms that record the context of experimental measurements.

2. Provides a mechanism for associating elements of the experimental design with standard ontology terms. This annotation will promote interoperability and make the task of meta-analysis of experiments easier.

3. Provides the infrastructure for building interpretive assertions within reasoning models that can trace their conclusions to the underlying data. The data can come directly from experiments or indirectly through the curation of published experimental reports. This can form the basis of a type of nanopublication that can trace its content to an underlying body of experimental data.

## Competing interests

The authors declare that they have no competing interests.

## Availability and Requirements

Software for the BioScholar project is described on the project home page at http://www.bioscholar.org/. The source code and applications are hosted at our google code project webpage http://code.google.com/p/bioscholar. This includes a non-editable implementation of the neural connectivity knowledge base (that may be installed in an easy, one-click step) as well as a functional version of the general BioScholar system. This software is distributed under the MIT Open Source License. Running the self-contained server code requires Java 1.6 or higher and a computer with 1GB RAM or more. The code is platform independent.

Running the web-based client requires a web browser with the Adobe^® ^Flash^® ^plugin, version 10 or higher.

## Authors' contributions

GAPCB formulated the basic idea behind the KEfED approach and coordinated the project. TAR and GAPCB developed the BioScholar web application and wrote the paper. CR and EH both contributed to the development of the 'Cycle of scientific Investigation' as the large-scale formulation into which KEfED modeling would apply. MB provided access to neural connectivity data and neuroanatomical ontologies from within the Brain Architecture Management System (BAMS).

## Supplementary Material

Additional file 1 **Description of the process of importing brain region containment data from the BAMS xml file for the Swanson 1998 atlas into PowerLoom**. Click here for file

Additional file 2 **Brain region information from the Swanson 1998 atlas as downloaded from the BAMS web site**. Click here for file

Additional file 3 **PowerLoom file defining the qualitative geometric relations between regions including PROPER-PART-OF and OVERLAPS**. This provides the basic vocabulary for describing the relation between atlas regions. Click here for file

Additional file 4 **PowerLoom file defining basic terms for representing an anatomical brain atlas**. Includes the BrainRegion concept and relations relating brain regions to their names and abbreviations. Click here for file

Additional file 5 **PowerLoom file containing the names and containment relationships between brain regions as defined in the Swanson 1998 brain atlas**. This is the file that contains the containment and name information from the BAMS xml file in PowerLoom format. Click here for file
