# Supplementary material for: Knowledge engineering tools for reasoning with scientific observations and interpretations: a neural connectivity use case
Source: BMC Bioinformatics. 2011 Aug 22;12:351. doi: 10.1186/1471-2105-12-351 (PMC3176268; doi:10.1186/1471-2105-12-351)
Supplement: Additional file 1 — Description of the process of importing brain region containment data from the BAMS xml file for the Swanson 1998 atlas into PowerLoom. [file 1471-2105-12-351-S1.PDF]

Importing the Swanson Atlas into PowerLoom

Reasoning about brain region containment in the BioScholar neural connectivity application requires using information from a neuroanatomical atlas. Since the tract tracing experiments used in that application were curated [1] using the Swanson 1998 brain atlas [2], that is the source of our brain region information. We obtained a description of the brain regions from the Brain Architecture Management System (BAMS) [3]as an xml file (`http://brancusi.usc.edu/bkms/xml/swanson-98.xml`) downloaded from the BAMS website [4].

The contents of the xml file were syntactically transformed into the PowerLoom® syntax [5], using relations from from a simple mereologic ontology using the relations *proper-part-of, overlaps, discrete* and *equal*. Axiomatization is limited to making *proper-part-of* transitive and symmetry for *overlaps* symmetric. (see the file `partof.plm`)

The syntactic transformation was straightforward. The portion of the BAMS xml encoding of the Swanson atlas which described the brain regions with elements such as

```
<part id="p526"
      name="Abducens nucleus proper"
      abbreviation="VI_p"
      is_part_of_idrefs="p524"
      url_base_ref="u1"
      url_param="aidi=526">
</part>
```

This entry identifies the brain region *Abducens nucleus proper* with ID *p526*, abbreviation *VI_p* which is part of another brain region with ID *p524*. The URL related entries can be used to reference this region at the BAMS web site. They are not currently used by the BioScholar system. This entry was transformed into PowerLoom syntax by a utility program. The transformation uses definitions from the file `atlas.plm` to define *BrainRegion* and the relations *name*, *abbreviation* and *url-name.* The resulting PowerLoom file is `swanson-1998.plm`.

```
(ASSERT (BrainRegion p526))
(ASSERT (= (name p526) "Abducens nucleus proper"))
(ASSERT (= (abbreviation p526) "VI_p"))
(ASSERT (/PART/PROPER-PART-OF p526 p524))
(ASSERT (url-name p526 "u1" "aidi=526"))
```

This provides BioScholar with a minimal encoding of the containment relationships between brain regions from the Swanson atlas.

Bibliography

1. Burns, G. A. (1997). *Neural Connectivity in the Rat: Theory, Methods and Applications.* PhD Thesis, Oxford University.

2. Swanson, L. W. (1998). Brain Maps: Structure of the Rat Brain (2 ed.). San Diego: Elsevier Academic Press.

3. Bota, M., Dong, H., & Swanson, L. (2005). The Brain Architecture Management System. Neuroinformatics , 3 (1), 15-48.

4. BAMS Main Page. (n.d.). Retrieved 2011, from The Brain Architecture Management System: http://brancusi.usc.edu/bkms/

5. Chalupsky, H., MacGregor, R. M., & Russ, T. (2010). PowerLoom® Manual. USC Information Sciences Institute.
